# Maxillary Labial Double Frenum Frenectomy Using a Diode Laser: A Case Report

**DOI:** 10.7759/cureus.63400

**Published:** 2024-06-28

**Authors:** Kaveri Paleriya, Ruchita Patil, Prasad Dhadse, Shrishti Salian, Sanehi Punse, Akansha Rai

**Affiliations:** 1 Department of Periodontics and Implantology, Sharad Pawar Dental College and Hospital, Datta Meghe Institute of Higher Education and Research, Wardha, IND

**Keywords:** midline diastema, case report, frenectomy, diode laser, double frenum

## Abstract

Maxillary labial frenectomy is a surgical procedure aimed at addressing midline diastema, where a strip of tissue creating a gap between two upper front teeth is removed. Typically, this strip extends from the vestibule of the gingivobuccal sulcus to the attached gingiva. The procedure is often performed using a diode laser, offering benefits including simplicity and safety for the patients. It can remarkably improve overall aesthetics and decrease the chances of gingival recession. This case report highlights the successful management of a female patient who arrived complaining of double frenum attachment leading to poor musculature support due to which she experiences poor aesthetics and hampered oral hygiene maintenance.

## Introduction

The maxillary labial frenum is a fold of connective tissue, mucous membrane, and occasionally muscle fibers. The frenum exhibits two distinct yet perplexing departures from the standard. One is a straightforward, benign growth, and the other is an actual abnormality. The former is by far the more typical of these. The usual frenum extends in the attached gingiva, but certain problems occur when the insertion is untypically placed [1]. There are four levels of abnormally placed frenum. The first one is mucosal, which attaches the frenum to a comparatively lower level. The second is a gingival attachment that is more likely joined to the gingival margins. The third is papillary attachment, in which the frenum is extended to the interdental papilla. The last and the most atypical type is papilla penetrating attachment, where the thick, broad fibrous tissue intrudes on the free gingiva and interferes with the normal function of the upper lip; it compromises aesthetics, gingival recession, and diastema formation, and hampers oral hygiene maintenance [2].

According to Sewerin's morphological types of the maxillary frenum, there are a total of eight categories despite of atypically placed frenum: simple frenum, persistent tectolabial frenum, simple frenum with an appendix, simple frenum with nodule, double frenum, frenum with nichum, bifid frenum, and wide frenum [3].

A survey was conducted in 2020; the cross-sectional study enrolled 400 patients comprising both males and females within the age group 20-40 years. After this, it is found that the prevalence rate is most common with simple frenum type (309 (77.44%)), followed by frenum with nodule (63 (15.75%)), frenum with an appendix and nichum (7 (1.75%)), wider frenum (6 (1.5%)), double frenum (3 (0.75%)), and bifid labial and persistent tectolabial frenum (2 (0.5%)). There was no significant difference between males and females [3,4]. This diverse morphological variation causes various abnormalities affecting one's overall facial profile. In this case, the patient presents with a double frenum leading to abnormal musculature, hence causing midline diastema [5].

## Case presentation

**Figure 1:**
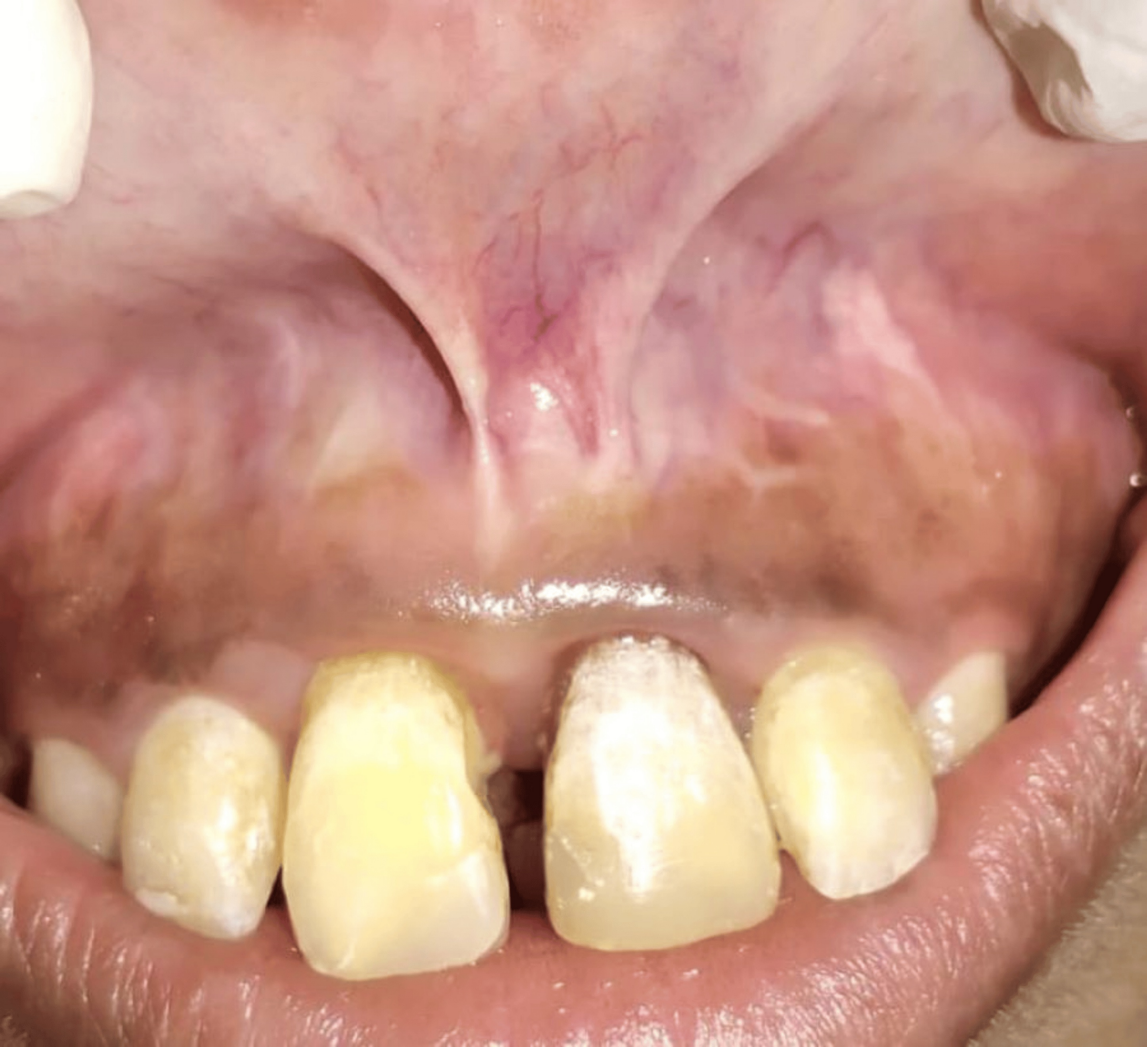
Preoperative photograph showing double frenum attachment with midline diastema

A 24-year-old female presented to the Department of Periodontics from the Department of Orthodontics with midline diastema as the abnormality leads to relapse and unfavorable outcomes of orthodontic treatment (Figure 1).

The patient had no significant medical history and was not on any immunosuppressive, antidiabetic, or antihypertensive drugs. The patient provided no relevant dental history. No gross asymmetry was observed during the extraoral examination, and there was bilaterally smooth and synchronized temporomandibular joint movement. The submental and submandibular lymph nodes on both sides were inspected, which were neither tender nor palpable.

After initial oral prophylaxis (ultrasonic scaling), the patient was then referred to the Department of Oral Pathology for blood investigations, including hemoglobin (Hb), bleeding time (BT), and clotting time (CT) (Table 1).

**Table 1:** Hematological reports Hb: hemoglobin, BT: bleeding time, CT: clotting time

Hematology	Finding value	Normal value
Hb %	12.5 g%	Male: 12-15.5 g%, female: 11-14.5 g%
BT	2 minutes and 10 seconds	1-3 minutes
CT	3 minutes and 5 seconds	1-5 minutes

**Figure 2:**
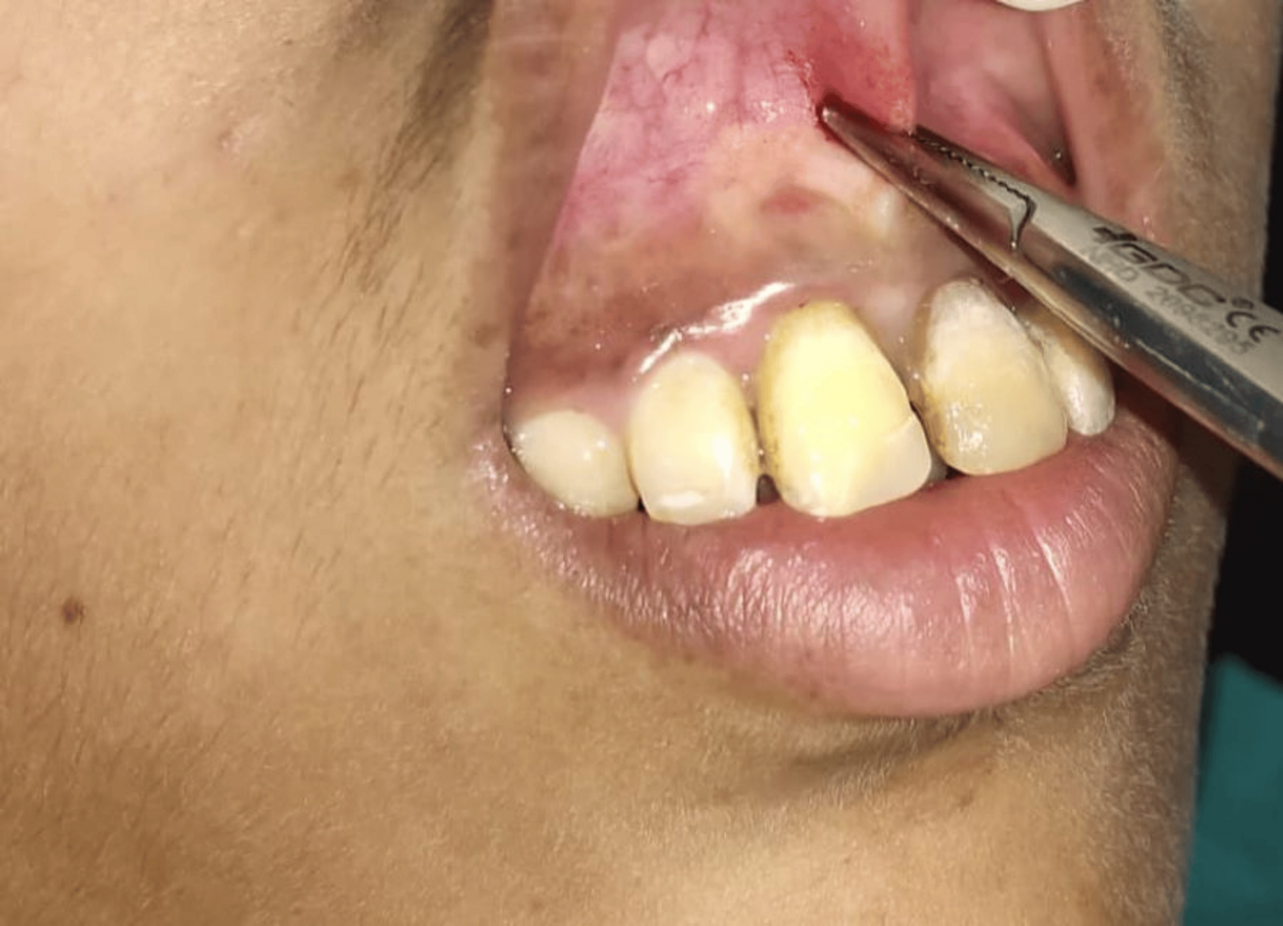
Frenum held using a hemostat

**Figure 3:**
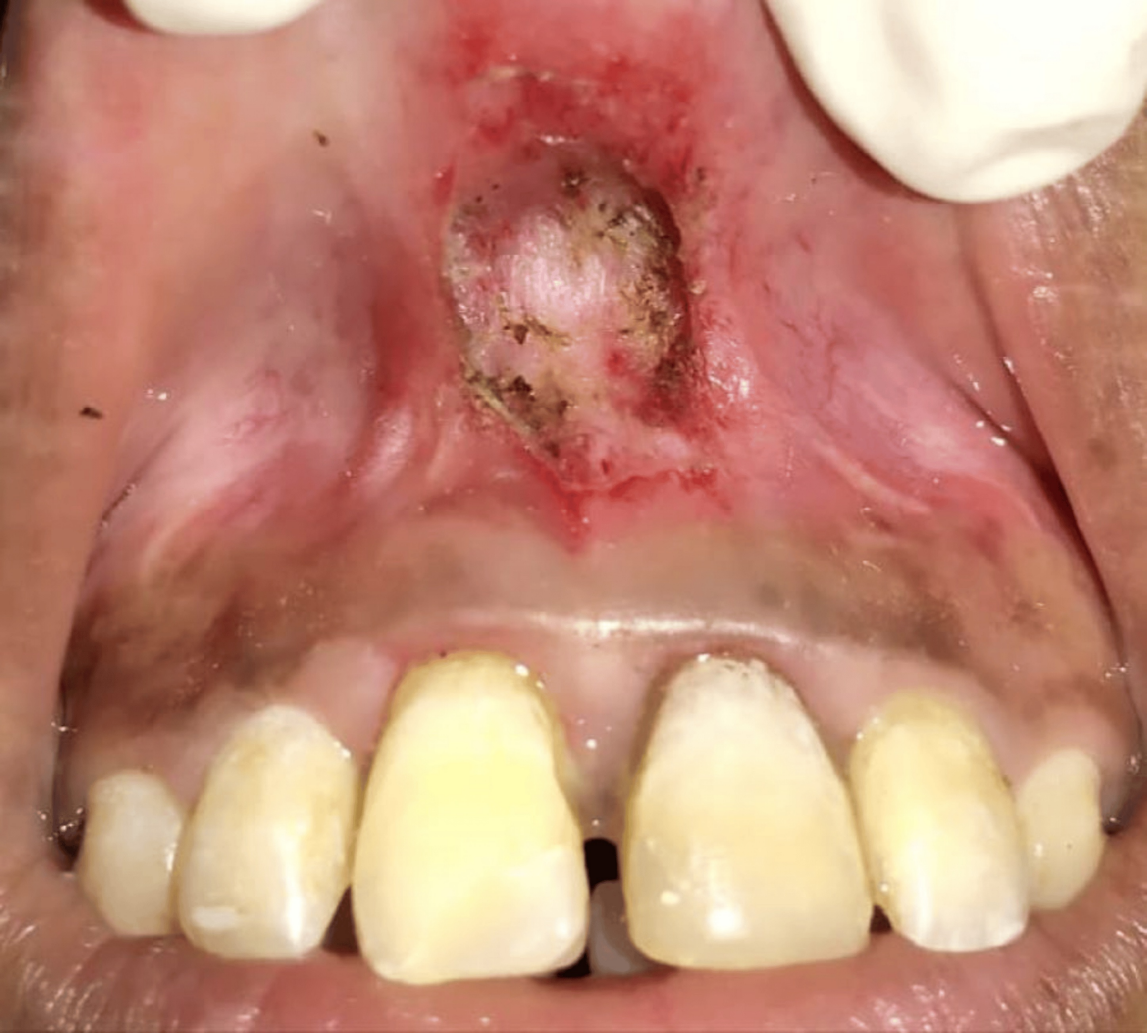
Frenectomy using laser

Under all aseptic conditions and precautions, local anesthesia was given; the labial frenum was held using a hemostat (Figure 2). A laser (EPIC X Diode Laser; Biolase, Foothill Ranch, CA) tip was then applied to the frenum vertically, and the fiberotomy was done (Figure 3).

**Figure 4:**
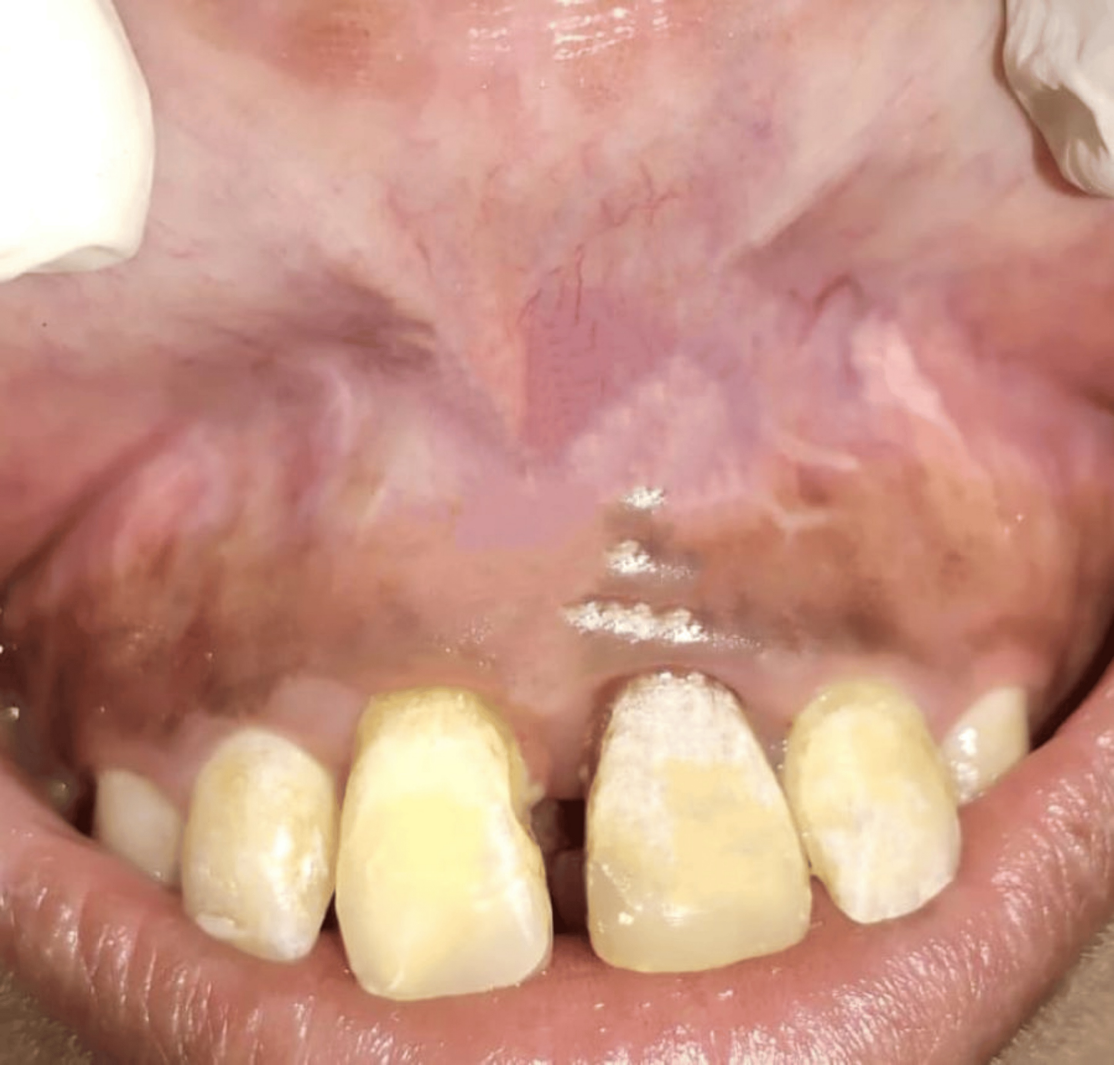
Postoperative healing after one month

After relieving fibrous attachment from the labial frenum, hemostasis was achieved. Following the surgery, the patient was provided with postoperative instructions. She was given a prescription for postoperative pain medication. A follow-up appointment was scheduled for re-evaluation after a week. Complete healing was seen after one month postoperatively (Figure 4).

## Discussion

One of the morphological variations of the frenum is the double labial frenum that can alter the normal musculature of the patient, leading to midline diastema, concerning poor aesthetics, which can not be possibly prevented or corrected by the means of orthodontic management as the case will result in relapse.

The surgical procedure involves two main methods of treatment: scalpel and laser. No dissimilarities were seen in the case of pocket depth, performing either of the methods. However, despite of desired outcome, there were several other factors that created huge differences between the methods chosen [6]. Many surgical procedures have been suggested by medical professionals. Some of these include procedures such as the scalpel method, frenectomy with soft tissue transplant, and frenal displacement with Z-plasty; however, there has been a new push to using lasers for frenectomy as laser therapy minimizes discomfort, trauma, and intervention time during a procedure. In this case, the application of a 980 nm diode laser is used, which proves to improve surgical accuracy and precision, minimizing needless tissue injury. This has been acknowledged by physicians and tolerated by patients [7,8]. Numerous studies have indicated that the laser procedure is preferable to the traditional knife method as the technique facilitates reduced operating time, excellent visualization, and a great hemostasis effect. The need for anesthesia is comparatively less, as well as chances of postoperative edema and pain, with minimal scarring, sterilization of wound site, and elimination of suturing [9]. Conversely, there were no negative impacts of the diode laser on the root surface. Therefore, it is safe to do diode laser surgery near dental hard tissue [10].

## Conclusions

In the presented case, the least complicated technique for treating a double labial frenum presented with diastema is laser-assisted frenectomy. It also demonstrates the finest possible outcome potential for minimally invasive dentistry. This research provides clinical evidence for the advantages of the diode laser. Maintaining a clean surgical field facilitates the surgery and reduces or eliminates postoperative pain. This procedure offers a rapid, safe, and efficient technique for both the patient and the surgeon.
